# The Nucleolar Localization Signal of Porcine Circovirus Type 4 Capsid Protein Is Essential for Interaction With Serine-48 Residue of Nucleolar Phosphoprotein Nucleophosmin-1

**DOI:** 10.3389/fmicb.2021.751382

**Published:** 2021-10-21

**Authors:** Jianwei Zhou, Yonghui Qiu, Ning Zhu, Linyi Zhou, Beining Dai, Xufei Feng, Lei Hou, Jue Liu

**Affiliations:** ^1^College of Veterinary Medicine, Yangzhou University, Yangzhou, China; ^2^Jiangsu Co-innovation Center for Prevention and Control of Important Animal Infectious Diseases and Zoonoses, Yangzhou University, Yangzhou, China

**Keywords:** porcine circovirus type 4, capsid protein, nucleolar localization signal, nucleolar phosphoprotein nucleophosmin-1, amino acid charge property

## Abstract

Porcine circovirus type 4 (PCV4) is an emerging etiological agent which was first detected in 2019. The nucleolar localization signal (NoLS) of PCV4 Cap protein and its binding host cellular proteins are still not elucidated. In the present study, we discovered a distinct novel NoLS of PCV4 Cap, which bound to the nucleolar phosphoprotein nucleophosmin-1 (NPM1). The NoLS of PCV4 Cap and serine-48 residue at the N-terminal oligomerization domain of NPM1 were necessary for PCV4 Cap/NPM1 interaction. Furthermore, the charge property of serine residue at position 48 of the NPM1 was crucial for its oligomerization and interaction with PCV4 Cap. In summary, our findings show for the first time that the PCV4 Cap NoLS and the NPM1 oligomerization determine the interaction of Cap/NPM1.

## Introduction

Porcine circoviruses (PCVs), which are small non-enveloped viruses within the genus *Circovirus* of the family *Circoviridae*, have a circular single-stranded DNA (ssDNA) genome with a size of about 1.7–2.0 kb in length (Breitbart et al., 2017). Four genotypes of PCVs (PCV1, PCV2, PCV3, and PCV4) have been recognized (Zhang et al., 2019; Oh and Chae, 2020; Opriessnig et al., 2020). PCV1 is not an etiological agent for pigs, but PCV2 infection results in a wide range of clinical symptoms, causing enormous economic loss to the global swine industries (Tischer et al., 1982; Gillespie et al., 2009). PCV3 was first detected in the United States in 2015 with metagenomic sequencing and is associated with various clinical diseases, including porcine dermatitis and nephropathy syndrome (PDNS), reproductive failure, respiratory disease, and diarrhea (Phan et al., 2016; Palinski et al., 2017; Jiang et al., 2019). PCV4, a previously unidentified PCV, which was first detected in Hunan province, China, in 2019, was considered to be related to serious clinical signs, including respiratory distress and PDNS (Zhang et al., 2019). Since then, PCV4 has also been discovered in some other provinces in China (Sun et al., 2020; Chen et al., 2021; Ha et al., 2021; Tian et al., 2021), demonstrating that PCV4 is probably prevalent in Chinese pig farms. Additionally, PCV4 was also reported in South Korea but was not discovered in Italy and Spain (Franzo et al., 2020; Nguyen et al., 2021).

The PCV4 genome contains a size of 1,770 nucleotides (nt) in length and encodes two major open reading frames (ORFs), namely, a replicase (Rep) and a capsid (Cap) gene (Zhang et al., 2019). Due to lack of autonomous DNA polymerases, circoviruses rely on the host cell’s replication machinery for viral DNA synthesis (Heath et al., 2006). For all PCVs, the N-terminal amino acids of the Cap protein constitute a nuclear localization signal (NLS) (Liu et al., 2001; Shuai et al., 2008; Mou et al., 2019). The NLS sequences, which consist of conserved amino acids, can be grouped into monopartite and bipartite motifs for transporting cellular proteins into the nucleus (Jans et al., 2000). The amino acid sequences of PCV4 Cap protein are remarkably distinct from those of PCV1, PCV2, and PCV3, but their motifs share high similarities within the NLSs regardless of different PCV genotypes (Liu et al., 2001; Shuai et al., 2008; Mou et al., 2019). The viral proteins containing NLS play a major role in viral genome replication, protein synthesis, and cell cycle progression and division (Wurm et al., 2001; Hiscox, 2002; Salvetti and Greco, 2014). As a newly identified virus with potentially evident implications on pig farming, the functions of PCV4 viral proteins are still not completely clear. Thus, mapping the functional NLS in PCV4 Cap will help us understand the function of the viral protein.

Nucleolar phosphoprotein nucleophosmin-1 (NPM1) participates in a number of cellular processes, such as DNA replication and repair, modulation of cell growth, ribosome biogenesis, and nucleocytoplasmic transport (Lindstrom, 2011). NPM1 is a multifaceted protein primarily situated in the nucleolus but frequently transporting between the nucleus and cytoplasm (Yun et al., 2003). Furthermore, NPM1 possesses a defined structure with functional domains containing an N-terminal oligomerization domain (OligoD), a central histone-binding domain (HistonD), and a C-terminal nucleic acid-binding domain (NBD) (Okuwaki, 2008). NPM1 has also been shown to involve various phases of viral infection by interacting with a wealth of viral proteins, such as human immunodeficiency virus type 1 (HIV-1) Rev (Fankhauser et al., 1991), human T-cell leukemia virus type 1 (HTLV-1) Rex (Adachi et al., 1993), adenoviral core (Samad et al., 2007), Japanese encephalitis virus (JEV) core (Tsuda et al., 2006), herpes simplex virus type 1 (HSV-1) UL24 (Lymberopoulos et al., 2011), Epstein–Barr virus nuclear antigen 2 (Liu et al., 2012), and capsid proteins of PCV2 and PCV3 (Zhou J. et al., 2020; Zhou J.W. et al., 2020; Song et al., 2021; Zhou et al., 2021). However, whether NPM1 binds to PCV4 Cap remains unknown.

In the present study, we reported that the nucleolar localization signal (NoLS) of PCV4 Cap and serine-48 residue of the NPM1 N-terminal oligomerization domain are required for PCV4 Cap/NPM1 interaction. Furthermore, the serine-48 charge property of the NPM1 is crucial for its oligomerization and interaction with PCV4 Cap. Overall, these results indicate for the first time that the NoLS of PCV4 Cap and oligomerization of the NPM1 determine Cap/NPM1 interaction.

## Results

### The Amino Acid Residues 1–37 at the N-Terminus of Porcine Circovirus Type 4 Cap Comprise a Nucleolar Localization Signal

**FIGURE 1:**
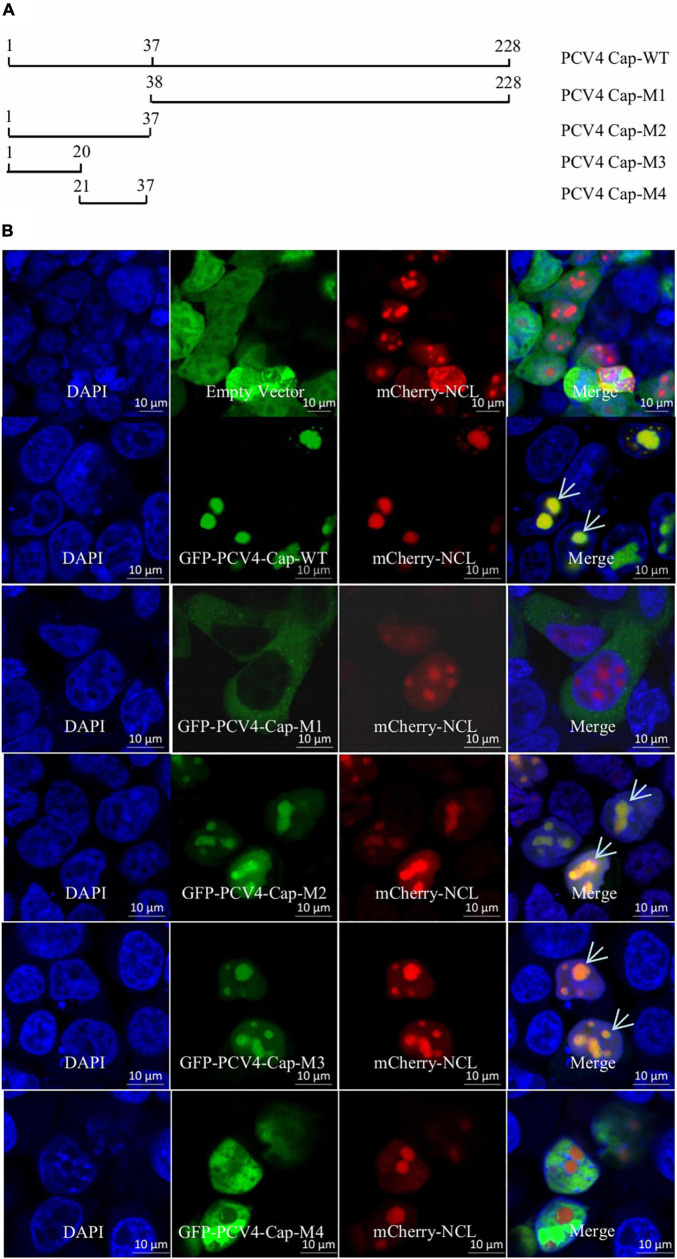
The N-terminal residues 1–37 of PCV4 Cap are a nucleolar localization signal. **(A)** Schematic depicting the truncated mutants of PCV4 Cap in the context. **(B)** HEK293T cells were co-transfected with mCherry-NCL and GFP-PCV4-Cap-WT, Cap-M1, Cap-M2, Cap-M3, or Cap-M4 for 24 h. The HEK293T cells were incubated with DAPI and then observed under a confocal microscope. Scale bar, 10 μm.

An Internet website (NucleOlar location sequence Detector^1^ and NLS Mapper^2^) was used to testify whether PCV4 Cap bears a NoLS. Luckily, a distinct novel NoLS might be present at the Cap N-terminal of all PCV4 strains deposited in GenBank. In order to confirm the NoLS, we constructed a huge range of PCV4 Cap-truncated mutants fused with GFP: Cap-WT (1–228 aa), Cap-M1 (38–228 aa), Cap-M2 (1–37 aa), Cap-M3 (1–20 aa), and Cap-M4 (21–37 aa) (Figure 1A). The indicated plasmids were, respectively, co-transfected into HEK293T cells together with the mCherry-nucleolin (NCL) plasmid. Confocal microscopy assays demonstrated that GFP-PCV4-Cap-WT, Cap-M2, and Cap-M3 could accumulate mostly in the nucleolus and colocalized with mCherry-NCL, while GFP-PCV4-Cap-M1 and Cap-M4 were located in the cytoplasm and nucleus, respectively (Figure 1B). The results indicated that amino acid residues 1–37 (mainly 1–20 aa) at the N-terminus of PCV4 Cap was a NoLS and played a prominent role in nucleolar localization.

### Porcine Circovirus Type 4 Cap Binds to Nucleolar Protein Nucleolar Phosphoprotein Nucleophosmin-1

**FIGURE 2:**
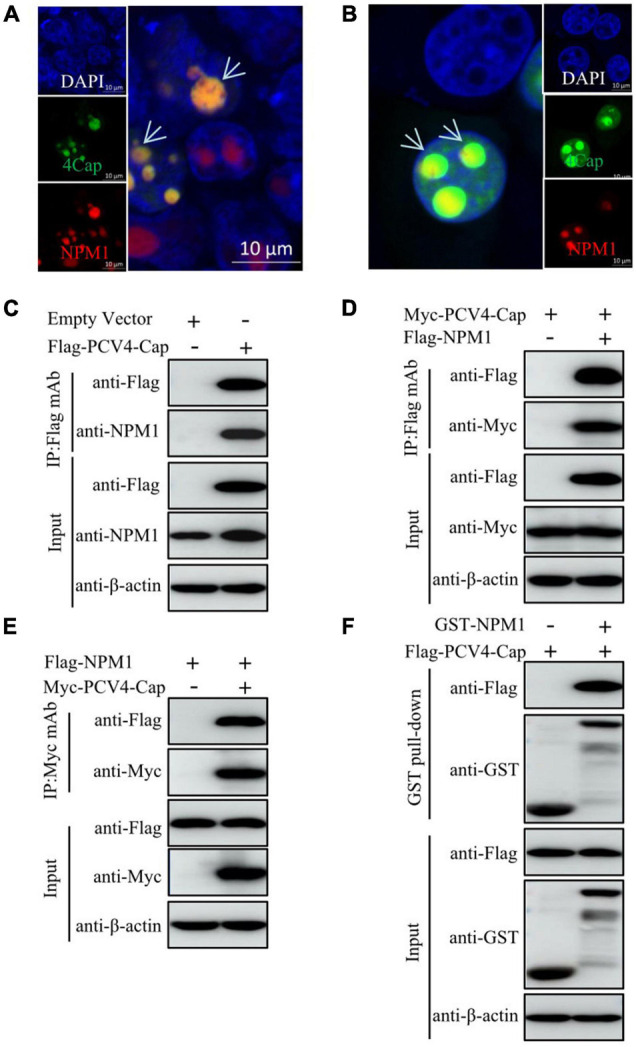
PCV4 Cap binds to NPM1. **(A,B)** Colocalization of NPM1 with PCV4 Cap in co-transfected cells. HEK293T cells **(A)** or PK-15 cells **(B)** were co-transfected with GFP-PCV4-Cap and mCherry-NPM1 for 24 h, and the cells were fixed followed by nuclei staining with DAPI **(A,B)**. Scale bar, 10 μm. **(C)** PK-15 cells were co-transfected with an empty vector and Flag-PCV4-Cap for 48 h. **(D,E)** The cell lysate extracts were immunoprecipitated with Flag beads **(C,D)** or anti-Myc purified IgG **(E)**. **(F)** The Flag-PCV4-Cap-transfected HEK293T cell lysates mixed with the GST, and GST-NPM1 proteins subjected to a GST pull-down assay and then detected by western blotting using corresponding antibodies.

As the NoLSs of Cap within different PCV genotypes exhibit high amino acid sequence homology and PCV2 and PCV3 Cap interact with NPM1 (Zhou J. et al., 2020; Zhou J.W. et al., 2020; Zhou et al., 2021), we continued to examine the intracellular localization of PCV4 Cap to NPM1. The results indicated that PCV4 Cap overlapped with NPM1 in the nucleoli of co-transfected HEK293T cells and PK-15 cells (Figures 2A,B). To further determine the relationship between PCV4 Cap and NPM1, co-immunoprecipitation (Co-IP) assays were conducted during transfection. We found that PCV4 Cap interacted with NPM1 in co-transfected HEK293T cells (Figures 2C–E). In addition, glutathione-*S*-transferase (GST) pull-down assays further confirmed that NPM1 interacted directly with PCV4 Cap (Figure 2F). These results showed that PCV4 Cap binds directly to NPM1.

### Amino Acid Residues 1–20 at the N-Terminus of Porcine Circovirus Type 4 Cap Are Essential for Association With Nucleolar Phosphoprotein Nucleophosmin-1

**FIGURE 3:**
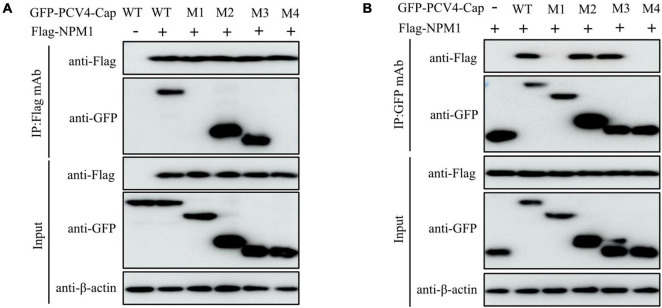
N-terminal residues 1–20 of PCV4 Cap are essential for binding to NPM1. **(A,B)** HEK293T cells were co-transfected with plasmids containing full-length PCV4 Cap or truncated mutants, together with the Flag-NPM1 plasmid for 48 h; the cell lysate extracts were immunoprecipitated with Flag beads **(A)** or anti-GFP purified IgG **(B)** and then detected by western blotting using the indicated antibodies.

To verify the domain within PCV4 Cap essential for binding to NPM1, plasmids GFP-PCV4 Cap-WT, Cap-M1, Cap-M2, Cap-M3, and Cap-M4 were, respectively, co-transfected into HEK293T cells along with the Flag-NPM1 plasmid. The results showed that amino acids (aa) 1–37 (M2) and 1–20 (M3) and the full-length PCV4 Cap (WT) could bind to NPM1, but aa 38–228 (M1) and aa 21–37 (M4) of Cap could not bind to NPM1 (Figures 3A,B). The results showed that the amino acid residues 1–20 at the N-terminus of Cap are essential for the interaction of PCV4 Cap with NPM1.

### Serine-48 in the Oligomerization Domain of Nucleolar Phosphoprotein Nucleophosmin-1 Is Required for Interaction With Porcine Circovirus Type 4 Cap

**FIGURE 4:**
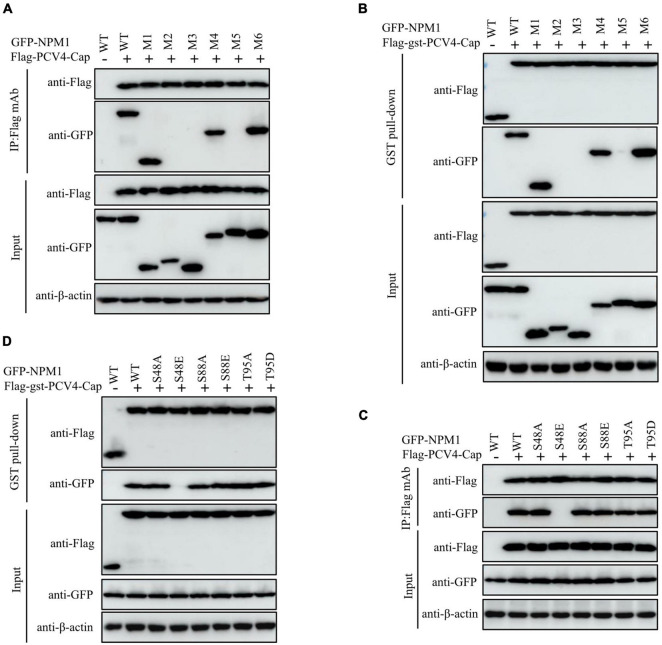
NPM1 serine-48 is crucial for binding to PCV4 Cap. **(A,B)** The NPM1-OligoD (1–117 aa) interacted with PCV4 Cap. HEK293T cells were co-transfected with expression plasmids GFP-NPM1-WT or its serial GFP-NPM1 truncated mutants M1 to M6, together with the Flag-PCV4-Cap or Flag-gst-PCV4-Cap plasmid. The cell lysate extracts were immunoprecipitated or subjected to a GST pull-down assay followed by western blotting using the indicated antibodies. **(C,D)** Mapping the critical amino acids of OligoD required for interaction with PCV4 Cap. HEK293T cells were co-transfected with NPM1 or NPM1 mutant plasmids (NPM1-S48A, NPM1-S48E, NPM1-S88A, NPM1-S88E, NPM1-T95A, or NPM1-T95D), together with the Flag-PCV4-Cap or Flag-gst-PCV4-Cap plasmid, and the cell lysate extracts were immunoprecipitated or subjected to a GST pull-down assay followed by western blotting using the indicated antibodies.

To testify that the domain in NPM1 is essential for interaction with PCV4 Cap, plasmids NPM1-WT (1–294 aa), NPM1-OligoD (1–117 aa), NPM1-HistonD (118–188 aa), NPM1-NBD (189–294 aa), NPM1-OligoD-HistonD (1–188 aa), NPM1-HistonD-NBD (118–294 aa), and NPM1-OligoD-NBD (1–117 aa + 189–294 aa) were, respectively, co-transfected into HEK293T cells together with the Flag-PCV4-Cap or Flag-gst-PCV4-Cap plasmid. The results demonstrated that the truncated mutants OligoD, OligoD-HistonD, and OligoD-NBD bound to PCV4 Cap, but HistonD, NBD, and HistonD-NBD did not (Figures 4A,B), demonstrating that the oligomerization domain of NPM1 is essential for interaction with PCV4 Cap.

Phosphorylation of the oligomerization domain of NPM1 is required for its structural polymorphism (Mitrea et al., 2014). To characterize the pivotal amino acid residues in the oligomerization domain of NPM1 responsible for binding to PCV4 Cap, we co-transfected a huge range of GFP-NPM1 mutants, containing GFP-NPM1-S48A, GFP-NPM1-S48E, GFP-NPM1-S88A, GFP-NPM1-S88E, GFP-NPM1-T95A, and GFP-NPM1-T95D, into HEK293T cells and conducted Co-IP and GST pull-down assays. We found that that the mutants NPM1-S48A, S88A, T95A, S88E, and T95D interacted with Flag-PCV4-Cap or Flag-gst-PCV4-Cap. However, the mimic-phosphorylated mutant S48E was not able to interact with PCV4 Cap (Figures 4C,D). Taken together, these results demonstrated that the unphosphorylated serine-48 of NPM1 was essential for PCV4 Cap/NPM1 interaction.

### Serine-48 Charge Property Plays a Vital Role in Nucleolar Phosphoprotein Nucleophosmin-1 Oligomerization and Its Interaction With Porcine Circovirus Type 4 Cap

**FIGURE 5:**
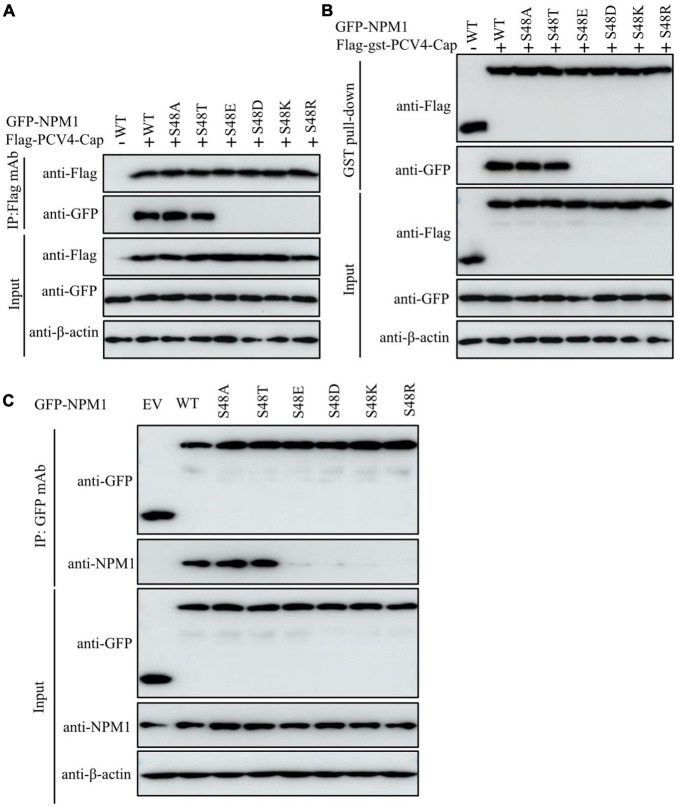
The serine-48 charge property of NPM1 determines its oligomerization and NPM1/Cap interaction. **(A,B)** The serine-48 charge property of NPM1 is essential for interaction with PCV4 Cap. HEK293T cells were co-transfected with NPM1 or NPM1 mutant plasmids (NPM1-S48A, NPM1-S48D, NPM1-S48E, NPM1-S48K, NPM1-S48R, or NPM1-S48T), together with the Flag-PCV4-Cap or Flag-gst-PCV4-Cap plasmid, and the cell lysate extracts were immunoprecipitated or subjected to GST pull-down assay followed by western blotting using the indicated antibodies. **(C)** Charged amino acids replaced by serine-48 disrupted the oligomerization of NPM1. Lysate extracts of PK-15 cells co-transfected with an empty vector, GFP-NPM1-WT, GFP-NPM1-S48A, GFP-NPM1-S48D, GFP-NPM1-S48E, GFP-NPM1-S48K, GFP-NPM1-S48R, or GFP-NPM1-S48T for 48 h were immunoprecipitated with anti-GFP purified IgG and then subjected to Co-IP assays.

The mutant NPM1-S48A but not the mutant NPM1-S48E bound to PCV4 Cap, which spurred us to further analyze the amino acid residue serine-48. The distinct charge properties existed between S48A and S48E; thus, we determined whether the serine-48 charge property of NPM1 was required for interaction with PCV4 Cap. Overexpression plasmids of GFP-NPM1-WT, GFP-NPM1-S48A, GFP-NPM1-S48D, GFP-NPM1-S48E, GFP-NPM1-S48K, GFP-NPM1-S48R, and GFP-NPM1-S48T were subjected to Co-IP and GST pull-down assays. We found that NPM1 bearing a neutral amino acid in serine-48 still bound to PCV4 Cap, but replacing the neutral amino acid with an acidic or basic one failed to interact with PCV4 Cap (Figures 5A,B). Therefore, these results indicated that the serine-48 charge property of NPM1 was essential for binding to PCV4 Cap.

To further characterize whether replacements of charged amino acids at serine-48 disrupted the oligomerization of NPM1 and then abolished NPM1/Cap interaction, we examined the interaction between GFP-tagged NPM1 mutants and endogenous NPM1 to test their ability to form oligomers. Lysate extracts of PK-15 cells after transfection with empty vector, GFP-NPM1-WT, GFP-NPM1-S48A, GFP-NPM1-S48D, GFP-NPM1-S48E, GFP-NPM1-S48K, GFP-NPM1-S48R, or GFP-NPM1-S48T for 48 h were immunoprecipitated with anti-GFP purified IgG and subjected to Co-IP assays. The results demonstrated that NPM1 bearing non-charged amino acids at serine-48 still interacted with endogenous NPM1, whereas switching of non-charged amino acids to charged ones reduced dramatically (Figure 5C). Hereby, we propose that charged amino acids replaced with serine-48 may alter the spatial conformation of NPM1 and disrupt the oligomerization of NPM1, which agreed with the previous study (Mitrea et al., 2014). Taken together, we concluded that the serine-48 charge property of NPM1 plays a major role in its oligomerization status and interaction with PCV4 Cap.

## Discussion

Porcine circovirus type 4 is an emerging etiological agent with enormous economic loss to global pig farms, which is linked to diverse clinical symptoms, including respiratory and gastrointestinal signs and PDNS (Zhang et al., 2019). Since 2019, PCV4 has been reported in about six provinces in China (Zhang et al., 2019; Sun et al., 2020; Chen et al., 2021; Ha et al., 2021; Tian et al., 2021). The PCV4 genome bears two ORFs: ORF1 encoding replicase and ORF2 encoding capsid (Zhang et al., 2019). The capsid protein belongs to the karyophilic proteins which are situated in the nucleus (Cheung and Bolin, 2002; Heath et al., 2006), and we found that PCV4 Cap could locate in the nucleolus as well (Figure 1B). Sequence analyses demonstrated that the N-terminus of Cap from different PCV genotypes contains highly conserved basic amino acids (Zhou J.W. et al., 2020). The intracellular distribution of truncated PCV4 Cap indicated that the N-terminal amino acid residues 1–37 of PCV4 Cap mediated the translocation of the capsid protein into the nucleus (Figure 1B). This is common to PCV1, PCV2, and PCV3 Cap (Liu et al., 2001; Shuai et al., 2008; Mou et al., 2019).

The NLSs are considered crucial components of viruses-encoding proteins (Wurm et al., 2001; Hiscox, 2002; Salvetti and Greco, 2014). Specific viral proteins containing NoLSs play a wide variety of roles in modulating cellular transcription and division as well as virus transcription and translation (Puviondutilleul and Christensen, 1993; Liu et al., 1997; Hiscox et al., 2001; Matthews, 2001; Wurm et al., 2001). No strict consensus sequences are observed on NLS, but the sequences are generally classified into monopartite or bipartite NLS (Silver, 1991). The motif of NoLS in PCV4 Cap (_8_RRRR-RR-RRR_20_) is similar to those identified in PCV1 Cap (_4_PRRR-RRRR-RPR-H_18_), PCV2 Cap (_4_PRRR-RRRRHRPR_18_), and PCV3 Cap (_8_RRR-R-RRRRRHRRR_22_) (Liu et al., 2001; Shuai et al., 2008; Mou et al., 2019). Synthesis of circovirus DNA occurs exclusively in the host cell nucleus, and the active nuclear import of ssDNA molecules requires karyophilic proteins (Heath et al., 2006). For PCVs and beak and feather disease virus, the NLS is essential for DNA accumulation (Cheung and Bolin, 2002; Heath et al., 2006). The N-terminus of PCV2 Cap can bind to receptor gC1qR on the nuclear membrane to modulate DNA binding (Fotso et al., 2016). This implies that the NLS of PCV4 Cap may contribute to DNA binding regulation of viral replication as well.

As a multifunctional nuclear protein, NPM1 takes part in multiple aspects of the viral life cycle, containing entry of the nucleus, viral genome synthesis, assembly of the capsid, and egress by interaction with viral NLS-containing proteins (Liu et al., 2012; Jeong et al., 2014; Day et al., 2015; Nouri et al., 2015). NPM1 is also a multifaceted protein bearing ribonuclease, molecular chaperone, and nucleic acid-binding activities (Okuwaki, 2008). The region binding to the core protein of Japanese encephalitis virus was mapped at the NPM1 N-terminus (Tsuda et al., 2006). The regions interacting with PCV2 and PCV3 Cap were also mapped at the NPM1 N-terminus, and serine-48 was indispensable for the interaction of PCV2 or PCV3 Cap with NPM1 (Zhou J. et al., 2020; Zhou J.W. et al., 2020; Zhou et al., 2021). Whether PCV4 Cap, bearing a previously unidentified NoLS, could interact with NPM1 and the role of PCV4 Cap in the viral replication have not been studied.

Our results showed that amino acid residues 1–37 at the N-terminus of PCV4 Cap comprise a NoLS (Figure 1). PCV4 Cap binds to nucleolar protein NPM1, and amino acid residues 1–20 at the N-terminus of PCV4 Cap are essential for association with NPM1 (Figures 2, 3). In addition, we demonstrated that the serine-48 charge property of NPM1 is crucial for its oligomerization and spatial conformation and binding to PCV4 Cap (Figures 4, 5). Intriguingly, when the serine-48 residue of NPM1 was mutated to neutral amino acids (Ala, Thr, etc.), it retained nucleolar localization. However, when it was mutated to acidic amino acids (Glu and Asp) or alkaline amino acids (Lys, Arg, and His), it no longer localized in the nucleolus (Zhou et al., 2021). Phosphorylation of the oligomerization domain is responsible for the spatial conformation of NPM1 and its nucleolar localization (Mitrea et al., 2014); we thus speculate that different charge properties of NPM1 affect its subcellular localization and interaction with other proteins and conclude that the serine-48 charge property of NPM1 is crucial for its oligomerization and spatial conformation and binding to PCV4 Cap (Figure 5). Considering that the NoLS at the N-terminus of PCV4 Cap functions as an NPM1 binding site, we hypothesize that NPM1 promotes intracellular nucleolar trafficking of PCV4 Cap. The NPM1 targets PCV4 Cap to the nucleolus *via* interaction with its NoLS and facilitates assembly of viral particles, and hence, it is essential for viral replication inside the nucleus of infected cells, which is consistent with the previous study (Duan et al., 2014).

At the early stage of PCV infection, Cap may enter the nucleolus for supporting viral transcription and genome DNA replication or disturbing the cell cycle progression of the S phase and synthesis of related cellular proteins (Finsterbusch et al., 2005). Accumulation of the viral proteins Rep, Rep’, and Cap in the nucleoplasm during PCV infection implied that encapsidation of the circular ssDNA and DNA replication occur exclusively in the nucleus but not in cytoplasmic compartments (Liu et al., 2001; Finsterbusch et al., 2005). It will be of great interest to further investigate whether Cap of PCV4 binds to other host factors for regulation of viral transcription.

In summary, our results mapped the NoLS of PCV4 Cap and showed that the serine-48 charge property of NPM1 played a vital role in its oligomerization and interaction with PCV4 Cap. Overall, this study broadens our insight into the nucleolar entry of PCV4 Cap and interaction with NPM1, thereby leading to highlighting potential targets for therapeutic intervention and prophylactic control of PCV4 infection.

## Materials and Methods

### Cells

PK-15 cells were cultivated in minimal essential medium (MEM) (Gibco, Carlsbad, CA, United States) supplemented with 10% fetal bovine serum (FBS) (Gibco). HEK293T cells (CRL-11268, ATCC, Manassas, VA, United States) were maintained in Dulbecco’s modified Eagle medium (DMEM) (Gibco). Both PK-15 and HEK293T cells were maintained in an incubator with 5% CO_2_ at 37°C.

### Antibodies and Reagents

Mouse monoclonal antibodies (mAbs) raised against GST (M0807-1) and β-actin (M1210-2) as well as rabbit polyclonal antibodies (pAbs) raised against Flag (0912-1), Myc (R1208-1), and GFP (SR48-02) were obtained from Huaan Biological Technology (Hangzhou, China). Mouse anti-Flag (F1804) and anti-Myc (05-419) mAbs were purchased from Sigma-Aldrich (St. Louis, MO, United States). Rabbit mAb raised against NPM1 (ab52644) was obtained from Abcam (Cambridge, MA, United States). Anti-Flag affinity resin (A2220) for immunoprecipitation was obtained from Sigma-Aldrich. NP-40 cell lysis buffer [50 mM Tris (pH 7.4), 150 mM NaCl, 1% NP-40] was obtained from Beyotime (P0013F; Shanghai, China). Horseradish peroxidase (HRP)-conjugated goat anti-rabbit and anti-mouse IgG were obtained from KPL (Milford, MA, United States).

### Construction and Transfection of Plasmid

DNA fragments containing full-length and truncated PCV4 Cap variants were amplified by polymerase chain reaction (PCR) from PCV4 genomic DNA (accession no. MK986820.1) (Zhang et al., 2019) and cloned into vectors pCMV-Flag-N, pCMV-Flag-gst-N, pCMV-Myc-N, or pEGFP-C3 (Clontech, Palo Alto, CA, United States) to obtain plasmids Flag-PCV4-Cap, Flag-gst-PCV4-Cap, GFP-PCV4-Cap-WT (1–228 aa), GFP-PCV4-Cap-M1 (38–228 aa), GFP-PCV4-Cap-M2 (1–37 aa), Myc-PCV4-Cap, GFP-PCV4-Cap-M3 (1–20 aa), and GFP-PCV4-Cap-M4 (21–37 aa). *NCL* (accession no. XM_021074959.1) and *NPM1* (accession no. XM_013990662.2) and its truncated *NPM1* variants from PK-15 cells were cloned into vectors pCMV-Flag-N, pmCherry-C1, pEGFP-C3, and pGEX-4T-1 (GE Healthcare Biosciences, Piscataway, NJ, United States) to obtain plasmids mCherry-NCL, mCherry-NPM1, Flag-NPM1, GFP-NPM1-WT (1–294 aa), GFP-NPM1-M1 (1–117 aa), GFP-NPM1-M2 (118–188 aa), GFP-NPM1-M3 (189–294 aa), GFP-NPM1-M4 (1–188 aa), GFP-NPM1-M5 (118–294 aa), GFP-NPM1-M6 (1–117 aa + 189–294 aa), GFP-NPM1-S88A, GFP-NPM1-S88E, GFP-NPM1-T95A, GFP-NPM1-T95D, GFP-NPM1-S48E, GFP-NPM1-S48D, GFP-NPM1-S48A, GFP-NPM1-S48T, GFP-NPM1-S48K, and GFP-NPM1-S48R. The primers used are summarized in Table 1. PK-15 or HEK293T cells grown onto plates or glass coverslips up to 70–90% confluency were transfected or co-transfected with 4.0 μg of the, respectively, indicated plasmids using the jetPRIME transfection reagent (Polyplus Transfection, New York, NY, United States) or ExFect transfection reagent (T101-01/02, Vazyme Biotechnology, Nanjing, China) as described in the manufacturer’s protocols.

**TABLE 1:** List of primers adopted in the study.

**Gene product**	**Sense primer (5′ to 3′)**	**Antisense primer (5′ to 3′)**
PCV4 Cap (1–228 aa)	ATGCCAATCAGATCTAGGTACA	TTATCCCTGTTTGGGGTAGTTAACA
PCV4 Cap (38–228 aa)	ATG CATGCGCGCTTCATGAGGGA	TTATCCCTGTTTGGGGTAGTTAACA
PCV4 Cap (1–37 aa)	ATGCCAATCAGATCTAGGTACA	TTA GTTCTTCCTTCTCCACCGGTATCTC
PCV4 Cap (1–20 aa)	TCGAGATGCCAATCAGATCTAGGTACAGCAGACGGAGGCGG AACCGGCGGAACCAGCGCAGGCGGTAAG	AATTCTTACCGCCTGCGCTGGTTCCGCCGGTTCCG CCTCCGTCTGCTGTACCTAGATCTGATTGGCATC
PCV4 Cap (21–37 aa)	TCGAGGGACTGTGGCCCCGGGCCAATAGGCGGAGATACCG GTGGAGAAGGAAGAACTAAG	AATTCTTAGTTCTTCCTTCTCCACCGGTATCTCCGC CTATTGGCCCGGGGCCACAGTCC C

### Confocal Microscopy

PK-15 or HEK293T cells co-transfected with 2.0 μg of the, respectively, indicated plasmids fused with mCherry or GFP tags for 24 h were fixed with 4% paraformaldehyde, stained with DAPI, and observed under a Nikon Al confocal microscope.

### Sodium Dodecyl Sulfate-Polyacrylamide Gel Electrophoresis and Western Blotting

The cell lysate extracts after transfection or co-transfection were collected and separated by standard sodium dodecyl sulfate-polyacrylamide gel electrophoresis (SDS-PAGE) and transferred to nitrocellulose membranes (GE Healthcare). After being blocked, the membranes were incubated with primary antibodies followed by being incubated with HRP-conjugated secondary antibodies. An enhanced chemiluminescence reagent (34096, Thermo Scientific) was used to visualize immunoreactive protein bands and further imaged by AI800 Images (GE Healthcare).

### Co-immunoprecipitation and Glutathione-*S*-Transferase Pull-Down Assays

For Co-IP assays, the cell lysate extracts from HEK293T cells co-transfected with 4.0 μg of the, respectively, indicated plasmids for 48 h were pretreated with protein A/G plus agarose (sc-2002, Santa Cruz Biotechnology), immunoprecipitated using anti-Flag beads, and then boiled before SDS-PAGE. For GST pull-down assays, expressed GST and GST-NPM1 were purified and immobilized on glutathione agarose beads (16100, Thermo Fisher Scientific, United States) to prepare the bait proteins, while Flag-PCV4-Cap-transfected HEK293T cell lysates served as the prey proteins. The detailed procedures for Co-IP and GST pull-down assays were performed as described elsewhere (Zhou J. et al., 2020; Zhou J.W. et al., 2020; Zhou et al., 2021).

## Data Availability Statement

All data generated for this study are included in the article.

## Author Contributions

JZ conceived, designed, and performed the experiments, interpreted the data and wrote the manuscript. JZ performed the experiments with assistance and advice from YQ, NZ, LZ, BD, XF, and LH. JL revised the manuscript. All authors have read and approved the submission.

## Conflict of Interest

The authors declare that the research was conducted in the absence of any commercial or financial relationships that could be construed as a potential conflict of interest.

## Publisher’s Note

All claims expressed in this article are solely those of the authors and do not necessarily represent those of their affiliated organizations, or those of the publisher, the editors and the reviewers. Any product that may be evaluated in this article, or claim that may be made by its manufacturer, is not guaranteed or endorsed by the publisher.

## Footnotes

1
http://www.compbio.dundee.ac.uk/www-nod/index.jsp

2
http://nls-mapper.iab.keio.ac.jp/cgi-bin/NLS_Mapper_form.cgi

## References

[B1] Adachi Y. Copeland T. D. Hatanaka M. Oroszlan S. (1993). Nucleolar targeting signal of Rex protein of human T-cell leukemia virus type I specifically binds to nucleolar shuttle protein B-23. *J. Biol. Chem.* 268 13930–13934. 8314759

[B2] Breitbart M. Delwart E. Rosario K. Segales J. Varsani A. Ictv Report C. (2017). ICTV virus taxonomy profile: circoviridae. *J. Gen. Virol.* 98 1997–1998. 10.1099/jgv.0.000871 28786778 PMC5656780

[B3] Chen N. H. Xiao Y. Z. Li X. S. Li S. B. Xie N. J. Yan X. L. (2021). Development and application of a quadruplex real-time PCR assay for differential detection of porcine circoviruses (PCV1 to PCV4) in Jiangsu province of China from 2016 to 2020. *Transbound. Emerg. Dis.* 68 1615–1624. 10.1111/tbed.13833 32931644

[B4] Cheung A. K. Bolin S. R. (2002). Kinetics of porcine circovirus type 2 replication. *Arch. Virol.* 147 43–58. 10.1007/s705-002-8302-4 11855635

[B5] Day P. M. Thompson C. D. Pang Y. Y. Lowy D. R. Schiller J. T. (2015). Involvement of nucleophosmin (NPM1/B23) in assembly of infectious HPV16 Capsids. *Papillomavirus Res.* 1 74–89. 10.1016/j.pvr.2015.06.005 27398412 PMC4934132

[B6] Duan Z. Q. Chen J. Xu H. X. Zhu J. Li Q. H. He L. (2014). The nucleolar phosphoprotein B23 targets Newcastle disease virus matrix protein to the nucleoli and facilitates viral replication. *Virology* 452, 212–222. 10.1016/j.virol.2014.01.011 24606698

[B7] Fankhauser C. Izaurralde E. Adachi Y. Wingfield P. Laemmli U. K. (1991). Specific complex of human immunodeficiency virus type 1 rev and nucleolar B23 proteins: dissociation by the Rev response element. *Mol. Cell Biol.* 11 2567–2575. 10.1128/mcb.11.5.2567 2017166 PMC360026

[B8] Finsterbusch T. Steinfeldt T. Caliskan R. Mankertz A. (2005). Analysis of the subcellular localization of the proteins Rep, Rep’ and Cap of porcine circovirus type 1. *Virology* 343 36–46. 10.1016/j.virol.2005.08.021 16168452

[B9] Fotso G. B. K. Bernard C. Bigault L. de Boisseson C. Mankertz A. Jestin A. (2016). The expression level of gC1qR is down regulated at the early time of infection with porcine circovirus of type 2 (PCV-2) and gC1qR interacts differently with the Cap proteins of porcine circoviruses. *Virus Res.* 220 21–32. 10.1016/j.virusres.2016.04.006 27063333

[B10] Franzo G. Ruiz A. Grassi L. Sibila M. Drigo M. Segales J. (2020). Lack of porcine circovirus 4 genome detection in pig samples from Italy and Spain. *Pathogens* 9:433. 10.3390/pathogens9060433 32486429 PMC7350368

[B11] Gillespie J. Opriessnig T. Meng X. J. Pelzer K. Buechner-Maxwell V. (2009). Porcine circovirus type 2 and porcine circovirus-associated disease. *J. Vet. Intern. Med.* 23 1151–1163. 10.1111/j.1939-1676.2009.0389.x 19780932 PMC7166794

[B12] Ha Z. Yu C. Xie C. Wang G. Zhang Y. Hao P. (2021). Retrospective surveillance of porcine circovirus 4 in pigs in Inner Mongolia, China, from 2016 to 2018. *Arch. Virol.* 166 1951–1959. 10.1007/s00705-021-05088-w 33987752

[B13] Heath L. Williamson A. L. Rybicki E. P. (2006). The capsid protein of beak and feather disease virus binds to the viral DNA and is responsible for transporting the replication-associated protein into the nucleus. *J. Virol.* 80 7219–7225. 16809327 10.1128/JVI.02559-05 PMC1489033

[B14] Hiscox J. A. (2002). The nucleolus – a gateway to viral infection? *Arch. Virol.* 147 1077–1089. 12111420 10.1007/s00705-001-0792-0 PMC7087241

[B15] Hiscox J. A. Wurm T. Wilson L. Britton P. Cavanagh D. Brooks G. (2001). The coronavirus infectious bronchitis virus nucleoprotein localizes to the nucleolus. *J. Virol.* 75 506–512. 11119619 10.1128/JVI.75.1.506-512.2001 PMC113943

[B16] Jans D. A. Xiao C. Y. Lam M. H. (2000). Nuclear targeting signal recognition: a key control point in nuclear transport? *Bioessays* 22 532–544. 10842307 10.1002/(SICI)1521-1878(200006)22:6<532::AID-BIES6>3.0.CO;2-O

[B17] Jeong H. Cho M. H. Park S. G. Jung G. (2014). Interaction between nucleophosmin and HBV core protein increases HBV capsid assembly. *FEBS Lett.* 588 851–858. 10.1016/j.febslet.2014.01.020 24462683

[B18] Jiang H. Wang D. Wang J. Zhu S. She R. Ren X. (2019). Induction of porcine dermatitis and nephropathy syndrome in piglets by infection with porcine circovirus type 3. *J. Virol.* 93:e02045–18. 10.1128/JVI.02045-18 30487279 PMC6363995

[B19] Lindstrom M. S. (2011). NPM1/B23: a multifunctional chaperone in ribosome biogenesis and chromatin remodeling. *Biochem. Res. Int.* 2011:195209. 10.1155/2011/195209 21152184 PMC2989734

[B20] Liu C. D. Chen Y. L. Min Y. L. Zhao B. Cheng C. P. Kang M. S. (2012). The nuclear chaperone nucleophosmin escorts an Epstein-Barr virus nuclear antigen to establish transcriptional cascades for latent infection in human B cells. *PLoS Pathog.* 8:e1003084. 10.1371/journal.ppat.1003084 23271972 PMC3521654

[B21] Liu J. L. Lee L. F. Ye Y. Qian Z. Kung H. J. (1997). Nucleolar and nuclear localization properties of a herpesvirus bZIP oncoprotein, MEQ. *J. Virol.* 71 3188–3196. 9060682 10.1128/jvi.71.4.3188-3196.1997 PMC191451

[B22] Liu Q. Tikoo S. K. Babiuk L. A. (2001). Nuclear localization of the ORF2 protein encoded by porcine circovirus type 2. *Virology* 285 91–99. 10.1006/viro.2001.0922 11414809

[B23] Lymberopoulos M. H. Bourget A. Ben Abdeljelil N. Pearson A. (2011). Involvement of the UL24 protein in herpes simplex virus 1-induced dispersal of B23 and in nuclear egress. *Virology* 412 341–348. 10.1016/j.virol.2011.01.016 21316727

[B24] Matthews D. A. (2001). Adenovirus protein V induces redistribution of nucleolin and B23 from nucleolus to cytoplasm. *J. Virol.* 75 1031–1038. 11134316 10.1128/JVI.75.2.1031-1038.2001 PMC113999

[B25] Mitrea D. M. Grace C. R. Buljan M. Yun M. K. Pytel N. J. Satumba J. (2014). Structural polymorphism in the N-terminal oligomerization domain of NPM1. *Proc. Natl. Acad. Sci. U.S.A.* 111 4466–4471. 10.1073/pnas.1321007111 24616519 PMC3970533

[B26] Mou C. Wang M. Pan S. Chen Z. (2019). Identification of nuclear localization signals in the ORF2 protein of porcine circovirus type 3. *Viruses* 11:1086. 10.3390/v11121086 31766638 PMC6950156

[B27] Nguyen V. G. Do H. Q. Huynh T. M. Park Y. H. Park B. K. Chung H. C. (2021). Molecular-based detection, genetic characterization and phylogenetic analysis of porcine circovirus 4 from Korean domestic swine farms. *Transbound. Emerg. Dis.* 00, 1–11. 10.1111/tbed.14017 33529468

[B28] Nouri K. Moll J. M. Milroy L. G. Hain A. Dvorsky R. Amin E. (2015). Biophysical characterization of nucleophosmin interactions with human immunodeficiency virus Rev and herpes simplex virus US11. *PLoS One* 10:e0143634. 10.1371/journal.pone.0143634 26624888 PMC4704560

[B29] Oh T. Chae C. (2020). First isolation and genetic characterization of porcine circovirus type 3 using primary porcine kidney cells. *Vet. Microbiol.* 241:108576. 10.1016/j.vetmic.2020.108576 31928694

[B30] Okuwaki M. (2008). The structure and functions of NPM1/Nucleophsmin/B23, a multifunctional nucleolar acidic protein. *J. Biochem.* 143 441–448. 10.1093/jb/mvm222 18024471

[B31] Opriessnig T. Karuppannan A. K. Castro A. M. M. G. Xiao C. T. (2020). Porcine circoviruses: current status, knowledge gaps and challenges. *Virus Res.* 286:198044. 10.1016/j.virusres.2020.198044 32502553

[B32] Palinski R. Pineyro P. Shang P. C. Yuan F. F. Guo R. Fang Y. (2017). A novel porcine circovirus distantly related to known circoviruses is associated with porcine dermatitis and nephropathy syndrome and reproductive failure. *J. Virol.* 91:e01879–16. 10.1128/JVI.01879-16 27795441 PMC5165205

[B33] Phan T. G. Giannitti F. Rossow S. Marthaler D. Knutson T. Li L. L. (2016). Detection of a novel circovirus PCV3 in pigs with cardiac and multi-systemic inflammation. *Virol. J.* 13:184. 10.1186/s12985-016-0642-z 27835942 PMC5105309

[B34] Puviondutilleul F. Christensen M. E. (1993). Alterations of fibrillarin distribution and nucleolar ultrastructure induced by adenovirus infection. *Eur. J. Cell Biol.* 61 168–176. 7693468

[B35] Salvetti A. Greco A. (2014). Viruses and the nucleolus: the fatal attraction. *Biochim. Biophys. Acta* 1842 840–847. 10.1016/j.bbadis.2013.12.010 24378568 PMC7135015

[B36] Samad M. A. Okuwaki M. Haruki H. Nagata K. (2007). Physical and functional interaction between a nucleolar protein nucleophosmin/B23 and adenovirus basic core proteins. *FEBS Lett.* 581 3283–3288. 17602943 10.1016/j.febslet.2007.06.024

[B37] Shuai J. Wei W. Jiang L. Li X. Chen N. Fang W. (2008). Mapping of the nuclear localization signals in open reading frame 2 protein from porcine circovirus type 1. *Acta Biochim. Biophys. Sin. (Shanghai)* 40 71–77. 10.1111/j.1745-7270.2008.00377.x 18180855

[B38] Silver P. A. (1991). How proteins enter the nucleus. *Cell* 64 489–497. 1991319 10.1016/0092-8674(91)90233-o

[B39] Song J. Hou L. Wang D. Wei L. Zhu S. Wang J. (2021). Nucleolar phosphoprotein NPM1 interacts with porcine circovirus type 3 Cap protein and facilitates viral replication. *Front. Microbiol.* 12:679341. 10.3389/fmicb.2021.679341 34113334 PMC8185148

[B40] Sun W. Du Q. Han Z. Bi J. Lan T. Wang W. (2020). Detection and genetic characterization of porcine circovirus 4 (PCV4) in Guangxi, China. *Gene* 773 145384. 10.1016/j.gene.2020.145384 33383119

[B41] Tian R. B. Zhao Y. Cui J. T. Zheng H. H. Xu T. Hou C. Y. (2021). Molecular detection and phylogenetic analysis of porcine circovirus 4 in Henan and Shanxi provinces of China. *Transbound. Emerg. Dis.* 68 276–282. 10.1111/tbed.13714 32634296

[B42] Tischer I. Gelderblom H. Vettermann W. Koch M. A. (1982). A very small porcine virus with circular single-stranded-DNA. *Nature* 295 64–66. 7057875 10.1038/295064a0

[B43] Tsuda Y. Mori Y. Abe T. Yamashita T. Okamoto T. Ichimura T. (2006). Nucleolar protein B23 interacts with Japanese encephalitis virus core protein and participates in viral replication. *Microbiol. Immunol.* 50 225–234. 10.1111/j.1348-0421.2006.tb03789.x 16547420

[B44] Wurm T. Chen H. Y. Hodgson T. Britton P. Brooks G. Hiscox J. A. (2001). Localization to the nucleolus is a common feature of coronavirus nucleoproteins, and the protein may disrupt host cell division. *J. Virol.* 75 9345–9356. 10.1128/Jvi.75.19.9345-9356.2001 11533198 PMC114503

[B45] Yun J. P. Chew E. C. Liew C. T. Chan J. Y. H. Jin M. L. Ding M. X. (2003). Nucleophosmin/B23 is a proliferate shuttle protein associated with nuclear matrix. *J. Cell Biochem.* 90 1140–1148. 10.1002/jcb.10706 14635188

[B46] Zhang H. H. Hu W. Q. Li J. Y. Liu T. N. Zhou J. Y. Opriessnig T. (2019). Novel circovirus species identified in farmed pigs designated as porcine circovirus 4, Hunan province, China. *Transbound. Emerg. Dis.* 67 1057–1061. 10.1111/tbed.13446 31823481

[B47] Zhou J. W. Dai Y. D. Lin C. Zhang Y. Feng Z. X. Dong W. R. (2020). Nucleolar protein NPM1 is essential for circovirus replication by binding to viral capsid. *Virulence* 11 1379–1393. 10.1080/21505594.2020.1832366 33073687 PMC7575006

[B48] Zhou J. Li H. Yu T. Li J. Dong W. Ojha N. K. (2020). Protein interactions network of porcine circovirus type 2 Capsid with host proteins. *Front. Microbiol.* 11:1129. 10.3389/fmicb.2020.01129 32582087 PMC7283462

[B49] Zhou J. Li J. Li H. Zhang Y. Dong W. Jin Y. (2021). The serine-48 residue of nucleolar phosphoprotein nucleophosmin-1 plays critical role in subcellular localization and interaction with porcine circovirus type 3 capsid protein. *Vet. Res.* 52:4. 10.1186/s13567-020-00876-9 33413620 PMC7792357

